# Methyl (*E*)-*N*′-[1-(2,4-dihydroxy­phen­yl)ethyl­idene]hydrazinecarboxyl­ate

**DOI:** 10.1107/S1600536809027317

**Published:** 2009-07-18

**Authors:** Lu-Ping Lv, Wei-Wei Li, Wen-Bo Yu, Tie-Ming Yu, Xian-Chao Hu

**Affiliations:** a Department of Chemical Engineering, Hangzhou Vocational and Technical College, Hangzhou 310018, People’s Republic of China; b Research Center for Analysis and Measurement, Zhejiang University of Technology, Hangzhou 310014, People’s Republic of China

## Abstract

The mol­ecule of the title compound, C_10_H_12_N_2_O_4_, adopts a *trans* configuration with respect to the C=N bond. The dihedral angle between the benzene ring and the methyl hydrazinecarboxyl­ate plane is 3.01 (6)°. An intra­molecular O—H⋯N hydrogen bond is observed. In the crystal, mol­ecules are linked into a two-dimensional network parallel to (10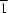) by O—H⋯O, N—H⋯O and C—H⋯O hydrogen bonds.

## Related literature

For general background to the properties of benzaldehyde­hydrazone derivatives, see: Parashar *et al.* (1988 ▶); Hadjoudis *et al.* (1987 ▶); Borg *et al.* (1999 ▶). For a related structure, see: Lv *et al.* (2008 ▶).
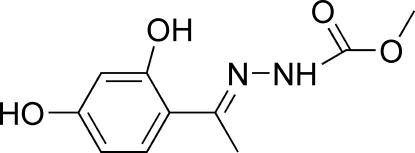


## Experimental

### 

#### Crystal data

C_10_H_12_N_2_O_4_
*M* *_r_* = 224.22
Monoclinic, 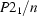

*a* = 10.714 (5) Å
*b* = 8.700 (4) Å
*c* = 11.682 (6) Å
β = 107.872 (6)°
*V* = 1036.3 (9) Å^3^
*Z* = 4
Mo *K*α radiation
μ = 0.11 mm^−1^
*T* = 223 K
0.24 × 0.23 × 0.19 mm

#### Data collection

Bruker SMART CCD area-detector diffractometer
Absorption correction: multi-scan (*SADABS*, Bruker, 2002 ▶) *T*_min_ = 0.977, *T*_max_ = 0.980
5096 measured reflections
1815 independent reflections
1355 reflections with *I* > 2σ(*I*)
*R*_int_ = 0.027

#### Refinement

*R*[*F* ^2^ > 2σ(*F* ^2^)] = 0.048
*wR*(*F* ^2^) = 0.131
*S* = 1.05
1815 reflections
149 parameters
H-atom parameters constrained
Δρ_max_ = 0.23 e Å^−3^
Δρ_min_ = −0.18 e Å^−3^

### 

Data collection: *SMART* (Bruker, 2002 ▶); cell refinement: *SAINT* (Bruker, 2002 ▶); data reduction: *SAINT*; program(s) used to solve structure: *SHELXS97* (Sheldrick, 2008 ▶); program(s) used to refine structure: *SHELXL97* (Sheldrick, 2008 ▶); molecular graphics: *SHELXTL* (Sheldrick, 2008 ▶); software used to prepare material for publication: *SHELXTL*.

## Supplementary Material

Crystal structure: contains datablocks I, global. DOI: 10.1107/S1600536809027317/ci2854sup1.cif

Structure factors: contains datablocks I. DOI: 10.1107/S1600536809027317/ci2854Isup2.hkl

Additional supplementary materials:  crystallographic information; 3D view; checkCIF report

## Figures and Tables

**Table 1:** Hydrogen-bond geometry (Å, °)

*D*—H⋯*A*	*D*—H	H⋯*A*	*D*⋯*A*	*D*—H⋯*A*
O2—H2⋯N1	0.82	1.84	2.557 (2)	145
O1—H1⋯O3^i^	0.82	1.98	2.791 (2)	170
N2—H2*B*⋯O2^ii^	0.86	2.35	3.185 (2)	164
C2—H2*A*⋯O3^i^	0.93	2.42	3.141 (3)	135

Symmetry codes: (i) 
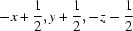
; (ii) 
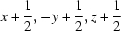
.

**Fig. 1.**
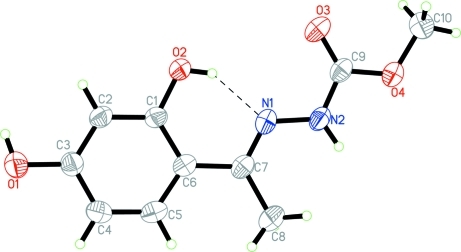
The molecular structure of the title compound, with the atom-numbering scheme. Displacement ellipsoids are drawn at the 40% probability level. The dashed line indicates a hydrogen bond.

**Fig. 2.**
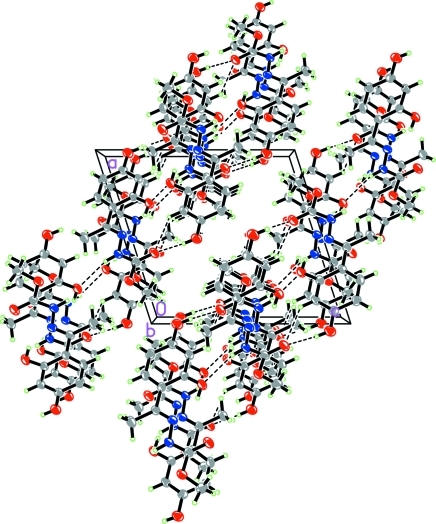
A packing diagram of the title compound. Hydrogen bonds are shown as dashed lines.

## supplementary crystallographic information

### Comment

Benzaldehydehydrazone derivatives have received considerable attention for a long time, due to their pharmacological activities (Parashar *et al.*, 1988) and their photochromic properties (Hadjoudis *et al.*,  1987). They are important intermidiates for 1,3,4-oxadiazoles, which have been reported to be versatile compounds with many properties (Borg *et al.*,  1999). As a further investigation of this type of derivatives, we report herein the crystal structure of the title compound.

The title molecule (Fig. 1) adopts a trans configuration with respect to the C═N double bond. The bond lengths and angles are comparable to those observed for (E)-methyl N'-[1-(4-methoxyphenyl)ethylidene]hydrazinecarboxylate (Lv *et al.*,  2008). The dihedral angle between benzene (C1-C6) and O3/O4/N1/N2/C7-C10 planes is 3.01 (6)°. An intramolecular O2—H2···N1 hydrogen bond is observed.

In the crystal structure, intermolecular O—H···O, N—H···O and C–H···O hydrogen bonds (Table 1) link the molecules into a two-dimensional network parallel to the (101) (Fig. 2).

### Experimental

2,4-Dihydroxy-acetophenone (1.52 g, 0.01 mol) and methyl hydrazinecarboxylate (0.90 g, 0.01 mol) were dissolved in stirred methanol (15 ml) and left for 2.5 h at room temperature. The resulting solid was filtered off and recrystallized from ethanol to give the title compound (yield 93%, m.p. 475-478 K). Single crystals of the title compound suitable for X-ray analysis were obtained by slow evaporation of an ethanol solution.

### Refinement

H atoms were positioned geometrically (N-H = 0.86 Å, O-H = 0.82Å and C-H =0.93 and 0.96 Å) and constrained to ride on their parent atoms with U_iso_(H) = xU_eq_(C,N), where x = 1.5 for methyl H and x = 1.2 for all other H atoms. A rotating group model was used for the methyl groups.

### Crystal data

**Table:**

C_10_H_12_N_2_O_4_	*F*(000) = 472
*M**_r_* = 224.22	*D*_x_ = 1.437 Mg m^−^^3^
Monoclinic, *P*2_1_/*n*	Mo *K*α radiation, λ = 0.71073 Å
Hall symbol: -P 2yn	Cell parameters from 1915 reflections
*a* = 10.714 (5) Å	θ = 2.3–25.0°
*b* = 8.700 (4) Å	µ = 0.11 mm^−^^1^
*c* = 11.682 (6) Å	*T* = 223 K
β = 107.872 (6)°	Block, colourless
*V* = 1036.3 (9) Å^3^	0.24 × 0.23 × 0.19 mm
*Z* = 4	

### Data collection

**Table:**

Bruker SMART CCD area-detector diffractometer	1815 independent reflections
Radiation source: fine-focus sealed tube	1355 reflections with *I* > 2σ(*I*)
graphite	*R*_int_ = 0.027
φ and ω scans	θ_max_ = 25.0°, θ_min_ = 2.3°
Absorption correction: multi-scan (*SADABS*, Bruker, 2002)	*h* = −12→12
*T*_min_ = 0.977, *T*_max_ = 0.980	*k* = −10→10
5096 measured reflections	*l* = −13→13

### Refinement

**Table:**

Refinement on *F*^2^	Primary atom site location: structure-invariant direct methods
Least-squares matrix: full	Secondary atom site location: difference Fourier map
*R*[*F*^2^ > 2σ(*F*^2^)] = 0.048	Hydrogen site location: inferred from neighbouring sites
*wR*(*F*^2^) = 0.131	H-atom parameters constrained
*S* = 1.05	*w* = 1/[σ^2^(*F*_o_^2^) + (0.0586*P*)^2^ + 0.2997*P*] where *P* = (*F*_o_^2^ + 2*F*_c_^2^)/3
1815 reflections	(Δ/σ)_max_ = 0.025
149 parameters	Δρ_max_ = 0.23 e Å^−^^3^
0 restraints	Δρ_min_ = −0.18 e Å^−^^3^

### Special details

**Table:**

Geometry. All esds (except the esd in the dihedral angle between two l.s. planes) are estimated using the full covariance matrix. The cell esds are taken into account individually in the estimation of esds in distances, angles and torsion angles; correlations between esds in cell parameters are only used when they are defined by crystal symmetry. An approximate (isotropic) treatment of cell esds is used for estimating esds involving l.s. planes.
Refinement. Refinement of F^2^ against ALL reflections. The weighted R-factor wR and goodness of fit S are based on F^2^, conventional R-factors R are based on F, with F set to zero for negative F^2^. The threshold expression of F^2^ > 2sigma(F^2^) is used only for calculating R-factors(gt) etc. and is not relevant to the choice of reflections for refinement. R-factors based on F^2^ are statistically about twice as large as those based on F, and R- factors based on ALL data will be even larger.

### Fractional atomic coordinates and isotropic or equivalent isotropic displacement parameters (Å^2^)

**Table:**

	*x*	*y*	*z*	*U*_iso_*/*U*_eq_	
O4	0.75341 (14)	0.02754 (18)	0.04159 (13)	0.0531 (4)	
O2	0.30274 (15)	0.34157 (19)	−0.17412 (12)	0.0549 (5)	
H2	0.3710	0.2990	−0.1355	0.082*	
O1	−0.00973 (15)	0.70770 (19)	−0.13812 (14)	0.0615 (5)	
H1	−0.0390	0.6873	−0.2100	0.092*	
O3	0.59381 (15)	0.1012 (2)	−0.12508 (13)	0.0592 (5)	
C6	0.32217 (19)	0.4395 (2)	0.02586 (17)	0.0403 (5)	
C7	0.44242 (19)	0.3525 (2)	0.08526 (18)	0.0426 (5)	
C2	0.1513 (2)	0.5234 (2)	−0.15377 (18)	0.0461 (5)	
H2A	0.1139	0.5203	−0.2369	0.055*	
C9	0.64512 (19)	0.1050 (2)	−0.01725 (18)	0.0443 (5)	
C1	0.26013 (19)	0.4342 (2)	−0.09982 (17)	0.0404 (5)	
C3	0.0972 (2)	0.6171 (2)	−0.08632 (19)	0.0458 (5)	
C5	0.2630 (2)	0.5360 (3)	0.09052 (19)	0.0501 (6)	
H5	0.3002	0.5422	0.1735	0.060*	
C4	0.1528 (2)	0.6219 (3)	0.03705 (19)	0.0522 (6)	
H4	0.1160	0.6829	0.0836	0.063*	
C10	0.8151 (2)	−0.0586 (3)	−0.0320 (2)	0.0615 (7)	
H10A	0.7509	−0.1217	−0.0874	0.092*	
H10B	0.8830	−0.1225	0.0184	0.092*	
H10C	0.8526	0.0113	−0.0760	0.092*	
C8	0.5058 (2)	0.3622 (3)	0.21894 (19)	0.0646 (7)	
H8A	0.5988	0.3485	0.2374	0.097*	
H8B	0.4708	0.2833	0.2577	0.097*	
H8C	0.4882	0.4611	0.2471	0.097*	
N2	0.60110 (16)	0.1850 (2)	0.06181 (15)	0.0469 (5)	
H2B	0.6418	0.1831	0.1377	0.056*	
N1	0.48811 (16)	0.2697 (2)	0.01548 (15)	0.0448 (5)	

### Atomic displacement parameters (Å^2^)

**Table:**

	*U*^11^	*U*^22^	*U*^33^	*U*^12^	*U*^13^	*U*^23^
O4	0.0468 (9)	0.0614 (10)	0.0448 (9)	0.0087 (7)	0.0047 (7)	0.0031 (7)
O2	0.0550 (9)	0.0643 (11)	0.0377 (8)	0.0175 (8)	0.0030 (7)	−0.0073 (7)
O1	0.0555 (10)	0.0627 (11)	0.0593 (10)	0.0148 (8)	0.0072 (8)	−0.0074 (8)
O3	0.0557 (9)	0.0700 (11)	0.0409 (9)	−0.0038 (8)	−0.0012 (7)	−0.0013 (8)
C6	0.0421 (11)	0.0408 (12)	0.0367 (11)	−0.0074 (9)	0.0102 (9)	0.0013 (9)
C7	0.0427 (11)	0.0445 (12)	0.0380 (11)	−0.0073 (9)	0.0084 (9)	0.0079 (9)
C2	0.0478 (12)	0.0482 (13)	0.0362 (11)	0.0001 (10)	0.0041 (9)	−0.0042 (9)
C9	0.0403 (11)	0.0470 (13)	0.0401 (12)	−0.0083 (10)	0.0044 (9)	0.0045 (10)
C1	0.0432 (11)	0.0401 (12)	0.0361 (11)	−0.0026 (9)	0.0096 (9)	−0.0024 (9)
C3	0.0423 (11)	0.0439 (12)	0.0489 (12)	−0.0008 (9)	0.0107 (10)	−0.0023 (10)
C5	0.0568 (13)	0.0568 (14)	0.0357 (11)	−0.0069 (11)	0.0127 (10)	−0.0018 (10)
C4	0.0574 (13)	0.0538 (14)	0.0482 (13)	0.0002 (11)	0.0202 (11)	−0.0081 (11)
C10	0.0548 (14)	0.0669 (16)	0.0635 (16)	0.0034 (12)	0.0191 (12)	−0.0028 (13)
C8	0.0664 (15)	0.0796 (18)	0.0404 (13)	0.0092 (13)	0.0053 (11)	0.0068 (12)
N2	0.0413 (10)	0.0562 (11)	0.0370 (9)	0.0021 (8)	0.0031 (8)	0.0065 (8)
N1	0.0377 (9)	0.0485 (11)	0.0435 (10)	−0.0013 (8)	0.0055 (7)	0.0078 (8)

### Geometric parameters (Å, °)

**Table:**

O4—C9	1.335 (2)	C2—H2A	0.93
O4—C10	1.445 (3)	C9—N2	1.352 (3)
O2—C1	1.362 (2)	C3—C4	1.381 (3)
O2—H2	0.82	C5—C4	1.375 (3)
O1—C3	1.369 (2)	C5—H5	0.93
O1—H1	0.82	C4—H4	0.93
O3—C9	1.210 (2)	C10—H10A	0.96
C6—C5	1.404 (3)	C10—H10B	0.96
C6—C1	1.414 (3)	C10—H10C	0.96
C6—C7	1.472 (3)	C8—H8A	0.96
C7—N1	1.291 (3)	C8—H8B	0.96
C7—C8	1.502 (3)	C8—H8C	0.96
C2—C3	1.378 (3)	N2—N1	1.378 (2)
C2—C1	1.381 (3)	N2—H2B	0.86
			
C9—O4—C10	116.06 (17)	C4—C5—H5	118.4
C1—O2—H2	109.5	C6—C5—H5	118.4
C3—O1—H1	109.5	C5—C4—C3	119.5 (2)
C5—C6—C1	115.61 (19)	C5—C4—H4	120.2
C5—C6—C7	121.90 (19)	C3—C4—H4	120.2
C1—C6—C7	122.48 (19)	O4—C10—H10A	109.5
N1—C7—C6	115.85 (18)	O4—C10—H10B	109.5
N1—C7—C8	123.37 (19)	H10A—C10—H10B	109.5
C6—C7—C8	120.8 (2)	O4—C10—H10C	109.5
C3—C2—C1	121.02 (19)	H10A—C10—H10C	109.5
C3—C2—H2A	119.5	H10B—C10—H10C	109.5
C1—C2—H2A	119.5	C7—C8—H8A	109.5
O3—C9—O4	124.6 (2)	C7—C8—H8B	109.5
O3—C9—N2	125.6 (2)	H8A—C8—H8B	109.5
O4—C9—N2	109.78 (17)	C7—C8—H8C	109.5
O2—C1—C2	116.30 (17)	H8A—C8—H8C	109.5
O2—C1—C6	122.56 (18)	H8B—C8—H8C	109.5
C2—C1—C6	121.14 (19)	C9—N2—N1	117.11 (17)
O1—C3—C2	121.94 (19)	C9—N2—H2B	121.4
O1—C3—C4	118.56 (19)	N1—N2—H2B	121.4
C2—C3—C4	119.5 (2)	C7—N1—N2	120.52 (17)
C4—C5—C6	123.2 (2)		
			
C5—C6—C7—N1	−178.96 (18)	C1—C2—C3—O1	179.24 (19)
C1—C6—C7—N1	−0.3 (3)	C1—C2—C3—C4	0.1 (3)
C5—C6—C7—C8	0.9 (3)	C1—C6—C5—C4	−0.6 (3)
C1—C6—C7—C8	179.6 (2)	C7—C6—C5—C4	178.17 (19)
C10—O4—C9—O3	3.7 (3)	C6—C5—C4—C3	−1.2 (3)
C10—O4—C9—N2	−177.44 (17)	O1—C3—C4—C5	−177.7 (2)
C3—C2—C1—O2	177.60 (19)	C2—C3—C4—C5	1.5 (3)
C3—C2—C1—C6	−2.0 (3)	O3—C9—N2—N1	−0.1 (3)
C5—C6—C1—O2	−177.40 (18)	O4—C9—N2—N1	−178.99 (16)
C7—C6—C1—O2	3.8 (3)	C6—C7—N1—N2	179.30 (16)
C5—C6—C1—C2	2.2 (3)	C8—C7—N1—N2	−0.5 (3)
C7—C6—C1—C2	−176.56 (19)	C9—N2—N1—C7	−178.00 (18)

### Hydrogen-bond geometry (Å, °)

**Table:**

*D*—H···*A*	*D*—H	H···*A*	*D*···*A*	*D*—H···*A*
O2—H2···N1	0.82	1.84	2.557 (2)	145
O1—H1···O3^i^	0.82	1.98	2.791 (2)	170
N2—H2B···O2^ii^	0.86	2.35	3.185 (2)	164
C2—H2A···O3^i^	0.93	2.42	3.141 (3)	135

Symmetry codes: (i) −*x*+1/2, *y*+1/2, −*z*−1/2; (ii) *x*+1/2, −*y*+1/2, *z*+1/2.
